# Conceptual Framework for a New Tool for Evaluating the Quality of Diabetes Consumer-Information Web Sites

**DOI:** 10.2196/jmir.5.4.e29

**Published:** 2003-11-27

**Authors:** Joshua J Seidman, Donald Steinwachs, Haya R Rubin

**Affiliations:** ^1^Center for Information TherapyHealthwise, IncWashington DCUSA; ^2^Department of Health Policy and ManagementJohns Hopkins UniversityBloomberg School of Public HealthBaltimore MDUSA; ^3^Departments of Medicine, Epidemiology, and Healthy Policy and ManagementJohns Hopkins School of Medicine and Bloomberg School of Public HealthBaltimore MDUSA

**Keywords:** Internet/standards, information management/standards, medical informatics/standards, guidelines, quality of health care, diabetes

## Abstract

**Background:**

Most existing tools for measuring the quality of Internet health information focus almost exclusively on structural criteria or other proxies for quality of information, rather than evaluating information accuracy and comprehensiveness.

**Objective:**

This research sought to build a conceptual framework that could lay the groundwork for a robust performance-measurement system for evaluating the quality of Internet health information.

**Methods:**

Application of the quality-of-care measurement paradigm to developing a conceptual framework for defining and evaluating the quality of diabetes consumer-information Web sites.

**Results:**

Performance measures related to accuracy and comprehensiveness of information can be added to structural criteria to provide a more-robust approach to Web site evaluation.

**Conclusions:**

The development and implementation of a reliable and valid method for evaluating the quality of Internet health sites could provide lay people with a tool to identify useful content more easily and distinguish between beneficial and misleading information.

## Introduction

It has become increasingly common for consumers to gather information on their own about medical care for themselves and their families. In the last few years, the increasing involvement of consumers in medical care decisions has dovetailed with the explosion of the World Wide Web as an accessible information source. These 2 forces theoretically have the power to reshape the organization and delivery of modern medical care by reducing the enormous asymmetry that exists between patients and their doctors. That metamorphosis cannot transpire, however, unless lay people can access reliable, accurate information in a digestible form. Mark Twain said "a lie can travel halfway around the world while the truth is putting on its shoes" (in an era when the fastest means of long-distance communication was the Pony Express). With the Internet, misinformation can travel around the world multiple times and potentially adversely affect many people's lives.

Despite the proliferation of health care Web sites little oversight of health care content exists, and no widely-accepted method for evaluating the quality of health and medical information on the Internet has been integrated into the health care system. Although lay people have successfully found valuable information about their diseases on the Web, their ability to do so depends largely upon the particular condition, the sophistication of the consumers themselves, their access to resources, the amount of time they have available to gather information, and luck. In addition to the frustration of lay people unable to find understandable information as they struggle to handle a potentially-devastating condition, this process can produce other negative consequences. Without an adequate medical background, consumers may seize on misleading, incorrect, or oversimplified information that can be potentially harmful to them and enervating for their clinicians, because the latter often have to disabuse their patients of misinformation. Little research exists to document whether the Internet has directly caused harm—thus far, only a few anecdotal reports have been cited in the literature [1]—but Eysenbach and Kohler have initiated an online-database effort to collect such information [2].

The development and implementation of a valid method for evaluating the quality of Internet health sites could provide lay people with a tool to locate useful content more easily and have confidence the information is accurate and complete. Access to accurate and digestible information has the potential both to empower lay people and to raise the level of dialogue between them and their clinicians, thus enriching the patient-clinician relationship and ultimately improving the quality and efficiency of health care delivery.

### Impact of the Internet on the Health Care Delivery System

Never has the world of science and medicine been so immediately accessible to lay people. The Institute of Medicine report, Crossing the Quality Chasm, notes that the Web can bridge the chasm between doctor and patient and elevate their level of dialogue, allowing them to discuss diagnostic and treatment choices in a more sophisticated and timely manner [3]. Richer clinician-patient conversations preceded and followed by electronic educational tools offer an opportunity for sounder health care decision making, better information management, and more thorough and comprehensible disease management.

That potential, however, has by no means been realized, perhaps due in part to the inadequacies of the current state of available information. Although the health information available on the Internet may not be any different than the information that can be found through more traditional means, the sheer volume of it and the speed with which lay people can access it has implications for both its potential value and drawbacks [4]. As with other nascent technologies, little empirical research exists on the quality of information offered on the Web, but the early evidence suggests current health information is, to varying degrees, incomplete, inaccurate, oversimplified, and/or misleading [5- 11].

## Methods

### Process for Reviewing Existing Health-Information Web Site Evaluation Models

This literature review evaluates research and work presented not only in the traditional peer-reviewed literature, but also on the Internet. Several factors contribute to the reality that a majority of the work done in the area of the quality of health information on the Internet can be found on the Web and not in the peer-reviewed literature. First, given the embryonic stage of the subject, the speed to "publication" of the Web means that considerably less time has elapsed relative to the slower process of traditional peer-reviewed literature. Second, some would argue that the prevalence of commentary and review of the Web is much greater on the Internet than in paper, peer-reviewed literature. Third, evaluation of health Web sites crosses multiple academic disciplines and lay consumer interests, rendering it more appropriate in some senses for alternative distribution channels.

### Gathering Evaluation Criteria From Existing Models

Some studies to evaluate existing Web sites have already been conducted, although the body of evidence changes so rapidly that no review can be completely thorough or up to date (including this one). One of the more-recent systematic reviews was conducted by Eysenbach et al in 2002 [12], which assessed 79 distinct studies that met their inclusion criteria. As described in Table 1, included studies most frequently used technical criteria and accuracy, whereas completeness, design, and readability were employed to a considerably-lesser extent. There was enormous variation not only in the approaches used to assess criteria but also in the quality of the methodology in doing so.

We reviewed several other published tools and online instruments to identify both additional criteria and more-refined definitions [13- 21], with most criteria fitting into the categories described above. In many cases, authors listed criteria with minimal or no technical definition, leaving specification to each individual user of the system, vastly limiting capacity for standardized comparisons of Web sites by multiple users.

The desire to create empirical methods of Web site evaluation has led some researchers to experiment with the development of automated tools for health Web site evaluation. Price and Hersh [22] developed a computer program with the goal of assessing a site's likely relevance, likely credibility, likely bias, content, currency, and value of links. The rudimentary algorithms developed for this computer program were marginally successful in identifying clearly "undesirable" Web pages, but certainly could not provide a more-refined evaluation. Shon and Musen [23] found that even creating a rudimentary automated method for Web site evaluation was virtually impossible because many basic publishing elements were described on Web sites less than half the time: authorship (20%), attribution/references (32%), disclosure (41%), and currency (35%).

Some research has focused on the development of self-assessment methods for Web site evaluation, although few have attempted to evaluate these models. Jones [16] presented findings in 1999 on such a method, but the criteria used were highly subjective and therefore do not necessarily provide a useful tool for other users.

**Table 1:** Studies that met inclusion criteria of Eysenbach et al's systematic review [12]

**Criteria Group**	**What It Includes**	**Criteria**	
		**Number**	**%**
Technical criteria	Disclosures of authorship, ownership, sponsorship, advertising, dates, credentials, affiliations, or other. Provision of links, references, feedback mechanisms, contact information, or disclaimers. Explanation of sources, purpose, copyright, editorial review process, hierarchy of evidence, or balanced evidence. Ease of navigation and searching. Appropriate writing style (subjective).	53	67
Accuracy	Developed criteria prior to assessment. Evaluated claims without prior development of tool.	47	59
Completeness	Percentage of a priori-defined elements covered. Balance of information presented.	19	24
Design (aesthetics)	Visual aspect of site. Layout. Use of visual analog scale.	15	19
Readability	Use of Flesch-Kincaid or other readability formulas. Little attempt to assess comprehension.	11	14

One of the most-recent attempts to evaluate the quality of health Internet information comes from researchers sponsored by the European Union. The initial progress report issued by Eysenbach et al [24] in February 2001 indicates that they have chosen to define quality from a user perspective. Eysenbach et al explain, "We define 'quality' of a health Website (health information or e-health service) as the totality of properties (features and characteristics) that bear on its ability to satisfy stated or implied needs of the user." Eysenbach et al specifically reject the notion that some objective gold standard should be used to evaluate quality of health information. Rather, they argue, "Quality is not 'degree of excellence' in relation to some abstract concept, but is seen in relation to (and must be measured against) the needs and preferences of the users of the product or service." More recently, Eysenbach and Kohler conducted the first laboratory usability study and focus groups to describe consumer techniques for retrieval and assessment of Internet health information [25]. They found that consumers generally reported they could find the information they need despite suboptimal searching techniques and questionable reliance on subjective markers of health-information quality.

## Results

### Building a Conceptual Framework

Multiple approaches to understanding the impact of the Internet on health and health care could be employed to tackle this emerging field of research—perhaps one day to be dubbed *cyberology* or *eHealth services research*. Measuring the quality of health care offers a useful framework for conceptualizing the measurement of the quality of health information. After all, health care is, in part, an information business. With the exception of surgery and other invasive procedures, much of what happens in health care involves the exchange of information, although there are other aspects of communication that shape the patient-provider interaction [26- 27]. In fact, in many cases, the line between health care and health information remains blurred. To some extent, this has always been true, but there are reasons to believe that information will have increasing value in 21st-century delivery systems. The rapid adoption of Internet technology around the world has the potential to expand the capacity of health professionals to interact with their patients and provide patient information and monitoring across the Internet [3]. The Internet, therefore, offers opportunities but with caveats; the opportunity derives from the growth of a tool that allows people to communicate in ways that they always wanted, but that depends on appropriate information flowing to the parties in need of it.

This evolving notion of health-information quality adapted from the quality-of-care paradigm therefore provides the basis of a solid framework for evaluating the quality of health-information Web sites. Although high-quality health information generally is a prerequisite for quality health services, it does not guarantee effective care; it is a necessary but not sufficient condition. It is important to distinguish between the quality of the information itself and the quality of the use of that information. The latter basically reflects the quality of care. As a corollary, while image quality associated with MRI machines is necessary to ensure high-quality radiology care when that test is conducted, less-than-optimal use of that technology can result in poor quality even if the image quality is excellent. The difference, of course, is that consumers cannot access MRIs simply by sitting down at their home computers, but the Internet has helped to provide patients with an enormous amount of health information.

### The Quality-of-Care Measurement Framework

The field of quality-of-care measurement provides a solid foundation for understanding how to measure the quality of health information. Perhaps the most commonly-cited definition of quality of care is the one developed by the Institute of Medicine, which states that quality in health care is "the degree to which health services for individuals and populations increase the likelihood of desired health outcomes and are consistent with current professional knowledge" [28].

Donabedian [29] suggested that quality-of-care measures can be separated into 3 categories: structural measures, process measures, and outcome measures.

Structural measures address the underlying systems and infrastructure: are systems in place and are the right types of people assembled in the right way to allow for the provision of quality care? Accrediting bodies—such as the Joint Commission on Accreditation of Healthcare Organizations (JCAHO) [30], the National Committee for Quality Assurance (NCQA) [31], and the Utilization Review Accreditation Commission (URAC) [32]—have historically employed accreditation standards that address many structural factors, such as appropriate credentialing of physicians and evidence of effective quality-improvement projects. An example for diabetes might be determining whether a doctor has additional training or board certification in endocrinology or diabetes. Evidence has often been lacking that structural criteria actually relate to delivering better health care process, as defined below.

Process measures—such as NCQA's HEDIS (Health Plan Employer Data and Information Set) [33] measure of whether patients received an annual referral to an ophthalmologist for retinal screening—assess the extent to which health care providers have done the right things, that is, provided those specific treatments and behavior that have been proven to improve desired patient-health outcomes for similar patients. The value of a process measure depends on the strength of the evidence that links it with ultimate outcomes. Outcome measures, in contrast, address the end results of medical care (eg, for diabetic patients, symptoms, level of blood sugar or hemoglobin A1C achieved, vision, quality of life, or mortality) [34].

The advantages and limitations of process and outcome measures have been discussed elsewhere [35]. Briefly, structural and process measures are only as good as the evidence that relates them to health-outcome benefits for similar patients. However, evidence has not been gathered for all-important clinical situations, such as those with rare diseases or combinations of common conditions that have not been studied together.

On the other hand, outcomes are not feasible or valid in all situations. Many factors outside of health care providers' control affect patients' outcomes. If outcomes are to serve as measures of health care quality, they should be compared to outcomes for similar patients. Yet such risk adjustment or stratification techniques do not exist for many outcome measures or omit important factors (see HEDIS [33] for examples). For example, hemoglobin A1C levels in diabetes will vary depending on how well patients adhere to health advice and instructions. The entity being measured may have control over only a limited number of patient care factors or processes (eg, nonadherence, difficulty in affording medications, and other medical conditions); thus, outcome may be influenced by factors beyond the provider's or health plan's control. Transforming a limited evidence base into a body of health information for consumers involves challenges that are similar to those for transforming that modest evidence base into performance measures. This is why Donabedian expected that performance measures typically would be developed from the starting point of an evidence base, but generally would have to be supplemented by expert opinion [29].

A combination of structure, process, and outcome approaches may produce the best assessment of quality of care. For example, NCQA released a performance measure [36] in 1999 to assess cholesterol management after acute cardiovascular events through a process measure (whether a lipid profile was performed within one year after a heart attack or revascularization) linked with an intermediate outcome measure (whether the patient's low-density lipoprotein [LDL] cholesterol level was controlled to less than 130 mg/dL between 2 and 12 months following the event).

Performance indicators—be they process or outcome measures—provide quantitative feedback as to whether some quality-improvement intervention actually produced a desired change. Whereas a structural measure might ask whether the health plan targeted high-risk individuals and encouraged them to get their cholesterol measured, a performance measure actually gauges their performance in getting those people tested—even before the further step of specifically reducing their cholesterol levels.

One final way that Donabedian suggests for thinking about how measures serve different purposes is to contrast technology assessment with performance assessment. Whereas the former "are activities meant to determine the right things to do (or the right ways to behave)," performance measures are "meant to determine if the things known (or presumed) to be the right things to do (or the right ways to behave) have in fact occurred" [37].

This dichotomy between structural and process measures has particular relevance to the current state of the evaluation of health information on the Internet. Most of these efforts have exclusively included criteria focusing on the process by which information is developed; did the authors follow a process thought to increase the likelihood of producing accurate information (eg, peer review)? In other words, did the developers of information "behave" the right way? In contrast, little work has been done to evaluate the content of Web sites; for example, did the Web site actually produce information that was accurate and comprehensive? This shift offers more than a shift from structural to process criteria because it also has the potential to complement static, qualitative assessment with dynamic, quantitative measurement, much the same way NCQA has combined on-site accreditation (done once every 3 years) with annual HEDIS reporting of performance measures. Relative to Donabedian's quality-of-care measurement dichotomy, the performance measures would allow us to assess whether the right information has in fact been given to consumers.

### Development of a Systematic Approach to Web Site Evaluation

How can we put into operation this goal of applying performance measurement to assessment of the quality of information? The first step is to realize that, although a variety of Web site evaluation tools have been developed, virtually none of them derive from a scientific development process. The creation of qualitative evaluation systems, however, can be a scientific process if it relies on objective, systematic criteria that are applied in a consistent and reliable way.

A data abstraction tool that employs a defined set of reviewer criteria lays the foundation for an objective evaluation system to assess the credibility of health information on Web sites. The techniques can be deemed reliable if they can be consistently repeated to produce the same results. The techniques can be judged valid if they measure what they purport to measure.

### Translating the Quality-of-Care Conceptual Framework to Internet Health Information

Although quality-of-care measurement provides a useful framework for thinking about measuring the quality of health information, not all elements of that paradigm can be easily translated. Most importantly, as far as we are aware certain types of epidemiological and health-services research have never been conducted to answer specific questions regarding the impact of Internet health information on health outcomes. Ultimately, as with most other health interventions, one would want to know how specific types of Internet health information affect users in terms of health status, morbidity, and mortality. Although considerable research has been conducted to evaluate the impact of specific patient-education interventions on various outcomes—particularly in the areas of asthma [38- 42], diabetes [43- 44], and recovery from bypass surgery [45]—this research has involved structured and organized interventions. In some cases, these targeted interventions have involved self-care or self-management, but none of these studies specifically involved the Internet. It may be difficult to generalize findings from the existing literature to the less-structured, more-independent nature of Internet-based patient education.

The European Union has sponsored a group of researchers to create an "action plan for safer use of the Internet," [24] and in their first report on evaluating the quality of health information on the Web, Eysenbach et al addressed this issue of the relationship between health information and outcomes. They stated that the "ideal methodology to develop a reliable and valid instrument for evaluating Websites would be to start with some criteria with 'face validity,' applying these criteria to sites and comparing it to the health outcomes of people having used the site/service" [24]. In the same publication Eysenbach et al, however, point out that such an ideal is not currently possible and may never be so. They write, "Such a model does not exist, and the methodological challenges for creating such an instrument are huge (starting with the problem of determining the outcomes of patients)." They conclude: "It is questionable whether a reliable and valid instrument developed along these lines can ever exist."

**Figure 1:**
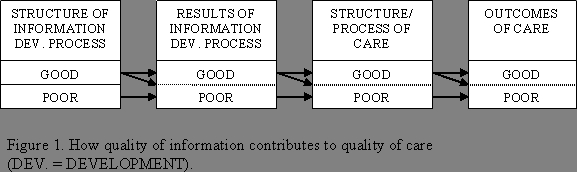
How quality of information contributes to quality of care

Despite this lack of available outcome research, Eysenbach et al [24] do not differentiate between what can be understood as 2 distinct notions, "the quality of health information" versus "the quality of health care." In contrast, the conceptual framework presented here specifically employs proxy measures to develop a systematic, measurable, objective method for evaluating Internet health-information quality. This method can still use some of the same principles from Donabedian's [29] structure-process-outcome paradigm. With respect to structural measures, one can assess whether the Web sites explain their methods for generating and updating health content, referencing sources, and instituting a peer-review process. Although health outcomes probably cannot be assessed, one can develop performance measures that address the outcomes of the health-information development process, in terms of the comprehensiveness and accuracy of the information provided compared with a gold standard. High-quality health information often is a prerequisite for high-quality care since information plays a critical role in most health care encounters. As shown in Figure 1, structural and process quality for information can often lead to good health information, which in turn can lead to high-quality health care processes, and ultimately to good health outcomes.

In Figure 1, good information-development processes are generally a necessary but not sufficient condition for producing information that truly is of high quality. That is, high-quality information processes can lead to either good or poor information, but poor processes will almost invariably lead to poor information quality. Similarly, a process that has performed well in producing high-quality information can lead to good health care processes, but poor information quality will virtually always result in poor care. One usually cannot achieve high-quality processes or outcomes of care without first having established that good results were achieved in the information-development process.

Definitions of quality may also vary depending on the objectives of those developing a particular Web site as well as the intentions of those seeking specific health information. Information may be high quality in terms of its accuracy and comprehensiveness but might not offer any therapeutic value if it does not drive user action or comfort the information seeker. In some instances, a consumer may already have all of the information he or she needs, but may seek a health Web site to assist with behavior change or emotional support. Such dimensions of quality in some ways move further down the chain of events to addressing the ability of a Web site to drive improvement in health outcomes. Future research should address these needs (see section on "Process for Creating a New Web Site Evaluation Model" below)—ideally, a user could go to one place to find both accurate-comprehensive information and support for behavior change goals and emotional needs—but they are somewhat distinct from the issues of whether the information itself is credible.

## Discussion

### The Current State of Web Site Evaluation and Oversight

Some evaluation methods have recently been developed to, theoretically, help consumers understand better what information they can trust. Aside from their providers, most consumers historically have relied principally on their friends and family to help them sort out health information; the Web has the effect of extending their community, thus allowing them to tap into a far-greater breadth of assistance, whether through static information, chat rooms, or online support groups. The evaluation models put forth to help consumers, however, have not been adequately tested, have not been adopted broadly, and do not have an infrastructure behind them that could support widespread implementation. A study in JAMA in 1998 by Jadad et al identified 47 Internet health-information rating tools and found that only 30% (14) offered a description of the criteria used, only 11% (5) provided instructions for their use, and none evaluated the interobserver reliability and construct validity of the measurements [46]. They concluded with a warning: "In summary, a large number of incompletely developed instruments to evaluate health information on the Internet exist. It is unclear, however, whether they should exist in the first place, whether they measure what they claim to measure, or whether they lead to more good than harm."

The authors updated their study 4 years later and found little change, except that many of the tools previously available no longer existed. Only 9 of the 47 rating instruments identified in 1997 continued to function. Of 51 newly-identified instruments, 11 were not functional, 35 were available but provided no information to allow for evaluation, and only 5 provided some information by which they could be evaluated. Furthermore, none of the 98 total instruments had been validated [47].

Petra Wilson suggests that tools designed to evaluate the quality of health information on the Internet can be broken down into 5 classifications: codes of conduct, quality labels, user guides, filters, and third-party certification [48]. These have different implications in terms of potential beneficiaries and the costs incurred by site providers, site users, and tool developers. Perhaps 2 of these efforts have garnered the most attention in the United States thus far: the Health on the Net Foundation has initiated a Code of Conduct, and users self-regulate and display the HONcode [49] (a complete listing of the criteria is in Appendix I of the cited reference); and Health Internet Ethics (Hi-Ethics) [50], whose standards have formed the basis of a new third-party Web site accreditation process overseen by URAC.

Organizations pledging to subscribe to the HONcode principles can post the HONcode icon on their Web pages. Although this effort at self-regulation offers a reasonable place to start, in terms of its ability to protect consumers from inaccurate and misleading information, it suffers from a variety of shortcomings. There are 3 overarching issues. First, the criteria are based on vague definitions; without specifications regarding how to evaluate individual sites, interpretation will vary dramatically. Second, the code relies solely on intent of the organization rather than actual performance; although intentionally-misleading information certainly seems more sinister and offends more from an ethical perspective, the damage done by inaccurate information is unrelated to whether it was offered with malice or by accident. One might expect that, regardless of how many organizations voluntarily adopt the HONcode, most health-information Web sites—if queried—probably would state that they abide by the underlying principles. Third, the policy relies entirely on self-policing; that is, the HONcode does not have any mechanism for auditing Web sites to assess whether they adhere to the code's principles.

The other recent self-regulation effort, Hi-Ethics, has broader goals that include protecting consumers' privacy concerns and addressing a range of other issues. As the organization's name implies, the Hi-Ethics principles focus more on ethical issues than health-information quality, although it developed a quality workgroup for its version 2.0 to allow for a more-intense examination of quality.

The first attempt at third-party Web site oversight was launched by URAC, which currently accredits an array of health plans and other health care organizations. URAC's standards, released in final form in July 2001, are based on the Hi-Ethics principles and are "intended for the accreditation of consumer-oriented Web-based electronic activities of health care organizations" [51]. The first 13 Web sites received URAC accreditation in December 2001. URAC's standards represent an important step forward, but they also have substantial limitations, as they are primarily designed to assess structural issues in Web site design and management and do not assess the specific quality or credibility of the information provided on the Web site.

Specifically, URAC's standards involve the following categories of standards: policies and procedures, quality oversight committee, disclosure, linking, privacy, security, accountability, and health content. However, this last category only addresses the Web site's policies and procedures for developing health content rather than any type of assessment of the content itself. Some of the issues addressed by URAC's standards—specifically, privacy and security—are extremely important but not directly related to the concept of health-information quality.

### Process for Creating a New Web Site Evaluation Model

No previous Web site evaluation models have specifically relied on a quality-of-care conceptual framework, and few have developed comprehensive and objective systems (based on a MEDLINE search on November 17, 2003). Probably the most-objective tool developed thus far was by RAND in an attempt to assess the quality of Web sites that provided information about breast cancer, depression, childhood asthma, and obesity [6], although this study only evaluated 10 sites per condition. However, many criteria offered by other health-services researchers, librarians, and Web commentators have merit. Therefore, the first step in developing a new model is to extract any valid and useful criteria from the review of the existing literature (and the Web itself) that are consistent with the quality-of-care conceptual framework.

The second step is to use the quality-of-care framework to identify critical gaps in existing systems—particularly with respect to objective criteria where current systems are most deficient—and add them to the model. One additional issue that remains to be resolved in the evaluation of health-information Web sites is that health information on any given Web site is not necessarily matched to the individual needs of that particular consumer. Ultimately, the system for Web site evaluation must assess practical aspects of computer access through a set of user-functionality criteria, which need to be assessed with research subjects (consumer users) actually navigating through the sites. One future approach to resolving this issue would be to develop a consumer/user survey that could be a component in the performance measurement portion of the evaluation tool, much the same way that the CAHPS (Consumer Assessment of Health Plans Study) survey has been integrated into HEDIS and NCQA's accreditation for evaluating health plan quality.

### Conclusion: Future Directions in the Conceptual Model

Although the focus of this research is to create the tool for creating performance measures of information quality, as stated early in the discussion of the conceptual model, this merely tackles the first step in understanding how the Internet can be used as a communication vehicle for influencing health. While its importance cannot be underestimated when a majority of Americans are accessing the Internet and 25 million of them used it in 2001 as a basis for making an important personal health care decision [52], we must remember that high-quality information represents only the beginning of the chain of effective communication.

Once one can assess the performance of the information-development process and know whether the input information is of high quality, health-services researchers can return to other conceptual frameworks to explore a range of communication problems. For example, communication theory [53] offers an important way to understand how we move along the quality-of-care paradigm as well. Because good information and effective communication are almost always important prerequisites in achieving good outcomes of care, we can benefit from learning more about how we move along the communication continuum from a sender's intended message to effective action on it by a receiver.

## Abbreviations

HEDIS: Health Plan Employer Data and Information Set
NCQA: National Committee for Quality Assurance
URAC: Utilization Review Accreditation Commission
